# Supplementary material for: Comparative genomic analysis of the PKS genes in five species and expression analysis in upland cotton
Source: PeerJ. 2017 Oct 30;5:e3974. doi: 10.7717/peerj.3974 (PMC5667535; doi:10.7717/peerj.3974)
Supplement: Table S1 [file peerj-05-3974-s001.docx]

**Table S1. Primers used in RT-PCR.**

| Primer name | Sequence (5' to 3') |
| --- | --- |
| *GhPKS1-F* | TTGGTCCTGGAATTACATT |
| *GHPKS1-R* | GCTGTTGGCATTATAAAGAT |
| *GhPKS2-F* | AGACATTGGTGCTTCGTA |
| *GHPKS2-R* | AATCTTCTCGTTCACATTCG |
| *GhPKS3-F* | GAATCCATCGTCAGAACAG |
| *GHPKS3-R* | TGGCATGTATAAGTTAATTTCAG |
| *GhPKS4-F* | GACCGTGGTGCTTCGTAG |
| *GHPKS4-R* | TTTCCGCCTTGTTTCCATTT |
| *GhPKS5-F* | CCGTGGTGCTTCATAGTATC |
| *GHPKS5-R* | AATTGTAGTGCAGCCAGG |
| *GhPKS6-F* | AGTGCCAAGAAGAAGAGA |
| *GHPKS6-R* | TTGAAGTTGTAAGACGTTATTC |
| *GhPKS7-F* | CTTACAGTGGAGACATTG |
| *GHPKS7-R* | CTCGTTCATATTCGCATA |
| *GhPKS8-F* | GAATACATGATAGAAGAGAT |
| *GHPKS8-R* | TATAATAGGAGATCCAACT |
| *GhPKS9-F* | GATTCGGTCCAGGTCTCA |
| *GHPKS9-R* | TTTGTCTGGGTCAGTTTGC |
| *GhPKS10-F* | CGTTGAGACGGTGGTTCT |
| *GHPKS10-R* | GATAACATGATGACGACGACTG |
| *GhPKS11-F* | CACTGTTGAGACTGTTGT |
| *GHPKS11-R* | GAGCACGACCAATTACTTA |
| *GhUBQ7-F* | GAAGGCATTCCACCTGACCAAC |
| *GhUBQ7-R* | CTTGACCTTCTTCTTCTTGTGCTTG |
