# Supplementary material for: Comparative genomic analysis of the PKS genes in five species and expression analysis in upland cotton
Source: PeerJ. 2017 Oct 30;5:e3974. doi: 10.7717/peerj.3974 (PMC5667535; doi:10.7717/peerj.3974)
Supplement: Table S2 [file peerj-05-3974-s002.docx]

**Table S2. The PKS genes identified in this study are listed.**

| Gene name | Gene ID | Chromo-some | AA | KD | No. Of exons | Preditced subcellular localization |
| --- | --- | --- | --- | --- | --- | --- |
| *GhPKS1* | CotAD_01172 | D5_chr10 | 386 | 42.6 | 2 | cyto |
| *GhPKS2* | CotAD_06418 | D5_chr11 | 393 | 43.6 | 2 | chlo |
| *GhPKS3* | CotAD_06419 | D5_chr11 | 332 | 36.1 | 2 | cyto |
| *GhPKS4* | CotAD_06433 | D5_chr11 | 386 | 42.2 | 2 | cyto |
| *GhPKS5* | CotAD_30473 | A2_chr9 | 393 | 43.8 | 2 | chlo |
| *GhPKS6* | CotAD_23550 | A2_chr8 | 397 | 43.9 | 2 | cyto |
| *GhPKS7* | CotAD_34322 | A2_chr11 | 393 | 43.7 | 2 | cyto |
| *GhPKS8* | CotAD_50293 | D5_chr8 | 396 | 43.7 | 2 | cyto |
| *GhPKS9* | CotAD_55273 | A2_chr6 | 329 | 35.9 | 2 | cyto |
| *GhPKS10* | CotAD_61611 | A2_chr8 | 349 | 38.1 | 2 | cyto |
| *GhPKS11* | CotAD_63043 | D5_chr11 | 394 | 43.3 | 2 | cyto |
| *AtPKS1* | AT1G02050.1 | At_Chr1 | 395 | 43.6 | 2 | cyto |
| *AtPKS2* | AT4G00040.1 | At_Chr4 | 385 | 42.4 | 2 | chlo |
| *AtPKS3* | AT4G34850.1 | At_Chr4 | 392 | 42.9 | 3 | cyto |
| *AtPKS4* | AT5G13930.1 | At_Chr5 | 395 | 43.1 | 2 | cyto |
| *MdPKS1* | MDP0000137655 | scaffold | 381 | 42.3 | 3 | cyto |
| *MdPKS2* | MDP0000208899 | Md_chr2 | 390 | 43.1 | 2 | cyto |
| *MdPKS3* | MDP0000257119 | Md_chr2 | 236 | 25.4 | 1 | cyto |
| *MdPKS4* | MDP0000287919 | Md_chr15 | 388 | 42.9 | 2 | cyto |
| *MdPKS5* | MDP0000302905 | Md_chr14 | 394 | 43.5 | 2 | cyto |
| *MdPKS6* | MDP0000432621 | Md_chr15 | 390 | 43.1 | 2 | cyto |
| *MdPKS7* | MDP0000575740 | Md_chr9 | 352 | 38.8 | 2 | cyto |
| *MdPKS8* | MDP0000641583 | Md_chr14 | 389 | 42.4 | 3 | cyto |
| *MdPKS9* | MDP0000686661 | Md_chr9 | 371 | 40.1 | 2 | cyto |
| *MdPKS10* | MDP0000686666 | Md_chr9 | 391 | 42.6 | 2 | cyto |
| *VvPKS1* | GSVIVT01010554001 | Vv_Chr16 | 344 | 37.5 | 3 | cyto |
| *VvPKS2* | GSVIVT01010556001 | Vv_Chr16 | 298 | 32.1 | 2 | cyto |
| *VvPKS3* | GSVIVT01010561001 | Vv_Chr16 | 256 | 27.3 | 2 | cyto |
| *VvPKS4* | GSVIVT01010565001 | Vv_Chr16 | 364 | 40.3 | 3 | cyto |
| *VvPKS5* | GSVIVT01010572001 | Vv_Chr16 | 427 | 47.3 | 3 | cyto |
| *VvPKS6* | GSVIVT01010574001 | Vv_Chr16 | 329 | 35.5 | 3 | nucl |
| *VvPKS7* | GSVIVT01010580001 | Vv_Chr16 | 344 | 37.6 | 2 | cyto |
| *VvPKS8* | GSVIVT01010581001 | Vv_Chr16 | 329 | 35.4 | 1 | cyto |
| *VvPKS9* | GSVIVT01010585001 | Vv_Chr16 | 392 | 42.7 | 1 | cyto |
| *VvPKS10* | GSVIVT01010590001 | Vv_Chr16 | 414 | 46.1 | 2 | cyto |
| *VvPKS11* | GSVIVT01018219001 | Vv_Chr15 | 284 | 31.3 | 2 | cyto |
| *VvPKS12* | GSVIVT01024107001 | Vv_Chr3 | 368 | 40.4 | 5 | cyto |
| *VvPKS13* | GSVIVT01032968001 | Vv_Chr13 | 334 | 35.8 | 3 | cyto |
| *PtPKS1* | Potri.014G145100.3 | Pt_Chr14 | 368 | 40.4 | 2 | cyto |
| *PtPKS2* | Potri.014G145100.2 | Pt_Chr14 | 396 | 43.3 | 2 | cyto |
| *PtPKS3* | Potri.014G145100.1 | Pt_Chr14 | 401 | 43.9 | 2 | cyto |
| *PtPKS4* | Potri.014G056300.1 | Pt_Chr14 | 389 | 42.5 | 2 | chlo |
| *PtPKS5* | Potri.012G138800.1 | Pt_Chr7 | 395 | 42.9 | 2 | cyto |
| *PtPKS6* | Potri.009G128800.1 | Pt_Chr9 | 391 | 42.6 | 3 | cyto |
| *PtPKS7* | Potri.005G175200.1 | Pt_Chr5 | 337 | 36.3 | 1 | cyto |
| *PtPKS8* | Potri.004G167300.1 | Pt_Chr4 | 392 | 42.5 | 2 | cyto |
| *PtPKS9* | Potri.003G176800.1 | Pt_Chr3 | 391 | 42.7 | 2 | cyto |
| *PtPKS10* | Potri.003G176700.1 | Pt_Chr3 | 391 | 42.4 | 2 | cyto |
| *PtPKS11* | Potri.002G141400.1 | Pt_Chr2 | 388 | 42.4 | 2 | cyto |
| *PtPKS12* | Potri.001G051600.1 | Pt_Chr15 | 390 | 42.5 | 2 | cyto |
| *PtPKS13* | Potri.001G051500.1 | Pt_Chr1 | 391 | 42.7 | 2 | cyto |
| *PtPKS14* | Potri.001G028600.1 | Pt_Chr1 | 376 | 41.6 | 2 | cyto |
