# Supplementary material for: Comparative genomic analysis of the PKS genes in five species and expression analysis in upland cotton
Source: PeerJ. 2017 Oct 30;5:e3974. doi: 10.7717/peerj.3974 (PMC5667535; doi:10.7717/peerj.3974)
Supplement: Table S3 [file peerj-05-3974-s003.docx]

**Table S3. Detailed information of the 20 motifs in the 52 PKS proteins.**

| Motif | Width | Sequences | Domain |
| --- | --- | --- | --- |
| 1 | 57 | EWGQPKSKITHLVFCTTSGVDMPGADYQLTKLLGLRPSVQRFMMYQQGCYAGGTVLR | Chal_sti_synt_N |
| 2 | 51 | HPGGPAILDQVEAKLGLKPEKLRATRHVLSEYGNMSSACVLFILDEMRKKS | Chal_sti_synt_C |
| 3 | 56 | LAKDLAENNKGARVLVVCSEITAVTFRGPSDTHLDSLVGQALFGDGAAAVIIGSDP | Chal_sti_synt_N |
| 4 | 49 | ERPLFQLVSAAQTFIPNSDGAIDGHLREMGLTFHLWRDVPQLISKNIEK | Chal_sti_synt_C |
| 5 | 35 | DKSMIKKRYMHMTEEILKENPNMCTYMAPSLDQRQ | Chal_sti_synt_N |
| 6 | 29 | KATTGEGLEWGVLFGFGPGLTVETVVLHS | Chal_sti_synt_C |
| 7 | 33 | PATILAIGTANPPNCVYQEDYPDYYFRITNCEH | Chal_sti_synt_N |
| 8 | 15 | FDPIGISDWNSLFWI | * |
| 9 | 15 | EVPKLGKEAALKCIK | * |
| 10 | 11 | TELKEKFKRMC | * |
| 11 | 15 | MVTVEEIRKAQRAQG | * |
| 12 | 29 | KRYIHLTEEMLEEHPNICAYMAPSLNIRQ | Chal_sti_synt_N |
| 13 | 21 | EWGLALAFGPGITFEGILLRS | Chal_sti_synt_C |
| 14 | 11 | MAPLVKDQVEP | * |
| 15 | 6 | FCHKLM | * |
| 16 | 19 | MSQTDNNGAPKHYATPTRR | * |
| 17 | 6 | FLTKTF | * |
| 18 | 15 | MMYEQICYAGDTVLR | * |
| 19 | 10 | METENNLEGC | * |
| 20 | 8 | PVEQTIYS | * |
