# Supplementary material for: Comparative genomic analysis of the PKS genes in five species and expression analysis in upland cotton
Source: PeerJ. 2017 Oct 30;5:e3974. doi: 10.7717/peerj.3974 (PMC5667535; doi:10.7717/peerj.3974)
Supplement: Table S4 [file peerj-05-3974-s004.docx]

**Table S4. Analysis of cis-acting elements of PKS gene promoter in upland cotton.**

|  | *GhPKS1* | *GhPKS2* | *GhPKS3* | *GhPKS4* | *GhPKS5* | *GhPKS6* | *GhPKS7* | *GhPKS8* | *GhPKS9* | *GhPKS10* | *GhPKS11* |
| --- | --- | --- | --- | --- | --- | --- | --- | --- | --- | --- | --- |
| ARE | 3 | 4 | 2 | 4 | 0 | 0 | 4 | 0 | 0 | 2 | 4 |
| ATCT-Motifs | 1 | 0 | 0 | 3 | 0 | 0 | 0 | 1 | 1 | 0 | 0 |
| Box I | 2 | 4 | 2 | 1 | 0 | 0 | 1 | 0 | 0 | 2 | 2 |
| Box III | 1 | 0 | 0 | 0 | 0 | 1 | 0 | 1 | 0 | 0 | 0 |
| Box 4 | 0 | 3 | 3 | 9 | 3 | 1 | 4 | 0 | 1 | 4 | 4 |
| G-Box | 0 | 2 | 0 | 3 | 4 | 1 | 2 | 3 | 4 | 7 | 9 |
| I-Box | 0 | 1 | 1 | 0 | 0 | 1 | 2 | 1 | 3 | 0 | 0 |
| CATT-Motifs | 1 | 1 | 0 | 0 | 2 | 2 | 0 | 1 | 1 | 0 | 0 |
| GT1-Motifs | 1 | 2 | 1 | 3 | 1 | 3 | 0 | 1 | 1 | 1 | 4 |
| HSE | 1 | 3 | 4 | 1 | 0 | 0 | 4 | 0 | 0 | 0 | 2 |
| MBS | 3 | 2 | 0 | 1 | 0 | 0 | 3 | 1 | 4 | 1 | 0 |
| MRE | 2 | 0 | 0 | 0 | 1 | 2 | 2 | 0 | 1 | 0 | 2 |
| TC-rich repeats | 3 | 1 | 1 | 1 | 1 | 1 | 2 | 2 | 2 | 0 | 1 |
| SP1 | 0 | 0 | 0 | 1 | 1 | 3 | 0 | 3 | 0 | 0 | 2 |
| CGTCA-Motifs | 1 | 0 | 0 | 0 | 1 | 2 | 0 | 2 | 0 | 0 | 0 |
