# Supplementary material for: Comparative genomic analysis of the PKS genes in five species and expression analysis in upland cotton
Source: PeerJ. 2017 Oct 30;5:e3974. doi: 10.7717/peerj.3974 (PMC5667535; doi:10.7717/peerj.3974)
Supplement: Table S5 [file peerj-05-3974-s005.docx]

**Table S5. Potential cis-elements in the 5’ regulatory sequences of the 11 *GhPKS* genes.**

| Site Name | Organism | Matrix score | Sequence | Function |
| --- | --- | --- | --- | --- |
| ARE | Zea mays | 6 | TGGTTT | cis-acting regulatory element essential for the anaerobic induction |
| ATCT-Motifs | Arabidopsis thaliana | 9 | AATCTAATCT | part of a conserved DNA module involved in light responsiveness |
| Box I | Pisum sativum | 7 | TTTCAAA | light responsive element |
| Box III | Pisum sativum | 10 | CATTTACACT | protein binding site |
| Box 4 | Petroselinum crispum | 6 | ATTAAT | part of a conserved DNA module involved in light responsiveness |
| G-Box | Pisum sativum | 6 | CACGTT | cis-acting regulatory element involved in light responsiveness |
| I-Box | Glycine max | 9 | GATAAGATA | part of a light responsive element |
| CATT-Motifs | Zea mays | 6 | GCATTC | part of a light responsive element |
| GT1-Motifs | Solanum tuberosum | 8 | AATCCACA | light responsive element |
| HSE | Brassica oleracea | 9 | AAAAAATTTC | cis-acting element involved in heat stress responsiveness |
| MBS | Zea mays | 6 | CGGTCA | MYB Binding Site |
| MRE | Petroselinum crispum | 7 | AACCTAA | MYB binding site involved in light responsiveness |
| TC-rich repeats | Nicotiana tabacum | 9 | GTTTTCTTAC | cis-acting element involved in defense and stress responsiveness |
| SP1 | Zea mays | 5 | CC(G/A)CCC | light responsive element |
| CGTCA-Motifs | Hordeum vulgare | 5 | CGTCA | cis-acting regulatory element involved in the MeJA-responsiveness |
