# Supplementary figures and images for: Comparative genomic analysis of the PKS genes in five species and expression analysis in upland cotton
Source: PeerJ. 2017 Oct 30;5:e3974. doi: 10.7717/peerj.3974 (PMC5667535; doi:10.7717/peerj.3974)

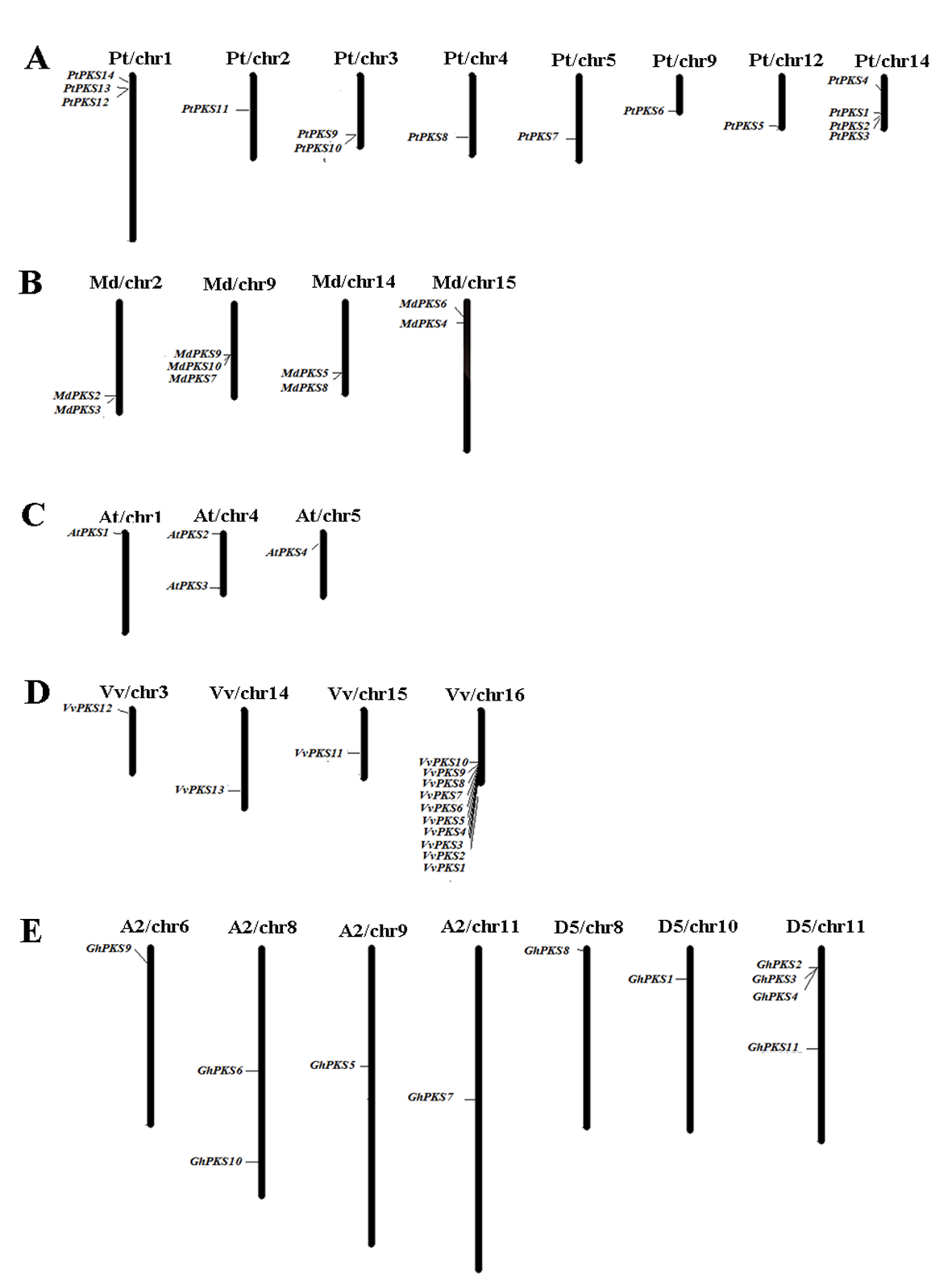

Supplement: Figure S1 — (A) Chromosome localization of 14 PKS genes in Populus tremula. (B) Chromosome localization of 10 PKS genes in Malus domestica. (C) Chromosome localization of 4 PKS genes in Arabidopsis thaliana. (D) Chromosome localization of 10 PKS genes in Vitis vinifera. (E) Chromosome localization of 11 PKS genes in Gossypium hirsutum. [file peerj-05-3974-s006.png]

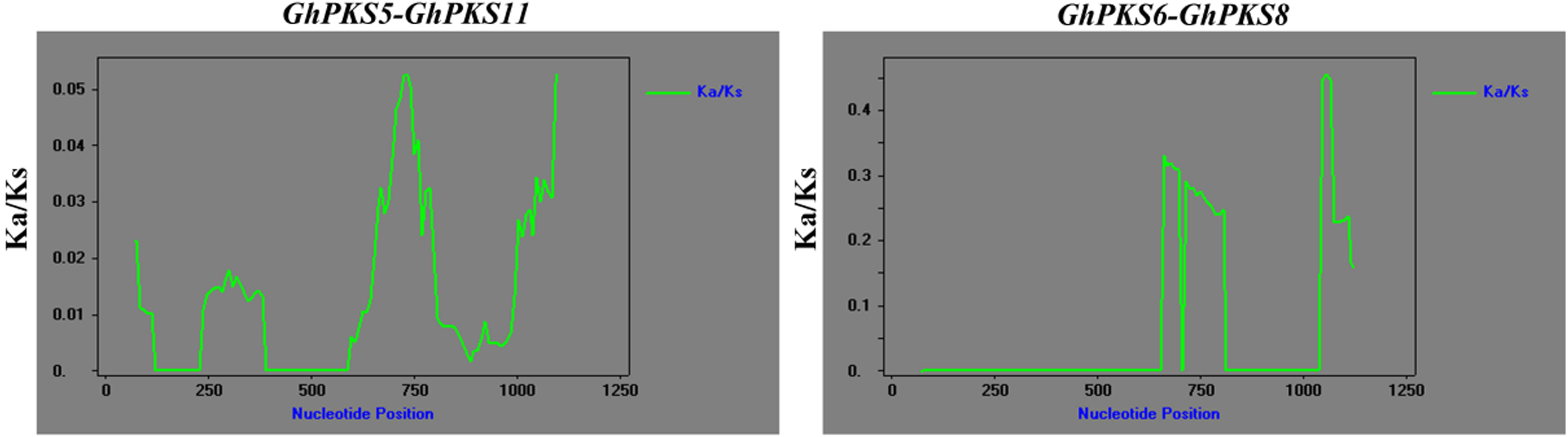

Supplement: Figure S2 [file peerj-05-3974-s007.png]
